# Quality of life and psychosocial well-being in thyroid eye disease in Japan: pre-teprotumumab national survey

**DOI:** 10.1210/jendso/bvag069

**Published:** 2026-03-27

**Authors:** Shun Emoto, Yoshito Bushita, Toshio Nagase, Kunihiro Nishimura, Yuji Hiromatsu, Yukiko Nishimura

**Affiliations:** NPO ASrid, Tokyo 113-0033, Japan; Value, Access & Policy, Amgen K.K., Tokyo 107-6239, Japan; Value, Access & Policy, Amgen K.K., Tokyo 107-6239, Japan; NPO ASrid, Tokyo 113-0033, Japan; Department of Diabetes and Endocrinology, Shin Koga Hospital, Fukuoka 830-8577, Japan; NPO ASrid, Tokyo 113-0033, Japan

**Keywords:** thyroid eye disease, quality of life, GO-QOL, SF-12, anxiety, depression

## Abstract

**Background:**

Thyroid eye disease (TED) causes functional and psychosocial morbidity, but little is known about the patient-experienced burden in Japan.

**Objective:**

To assess health-related quality of life (QOL) and psychological well-being among TED patients using standardized quantitative instruments and discuss patient-reported outcomes of patients in Japan compared to the United States, Europe, and Asia.

**Methods:**

This is the first Japanese nationwide cross-sectional survey that shows a natural history of QOL prior to the launch of teprotumumab outside of the United States. Fifty-six TED patients were recruited and answered questionnaires including the Short-Form 12-Item Survey version 2 scores (SF-12v2), Graves' Ophthalmopathy Quality of Life Questionnaire (GO-QOL), Hospital Anxiety Depression Scale, and Client Satisfaction Questionnaire 8. Disease activity and severity were classified based on European Group on Graves' Orbitopathy criteria.

**Results:**

While the SF-12v2 physical component summary and mental component summary scores were similar to population norms, SF-12v2 role component summary scores (mean = 39.5) were notably below the national normal. GO-QOL appearance-related/psychosocial scores were impaired as well as SF-12v2 and were particularly lower in active disease (52.5 vs 73.3; *P* = .004). The presence of symptoms such as pain with eye movement, eyelid swelling, strabismus, upper eyelid retraction, and proptosis was significantly associated with lower role/social functioning and satisfaction with care.

**Conclusion:**

TED imposes a significant psychosocial burden, particularly in active disease and among those with symptoms involving structure changes. Psychosocial support and targeted symptom control may improve patient-centered outcomes in TED.

Thyroid eye disease (TED) is an autoimmune disorder of the orbit, frequently associated with Graves' disease and chronic thyroiditis [1]. The disease is characterized by an inflammatory cascade, mediated in part by the interaction between the TSH receptor and IGF-1 receptor, which causes expansion of orbital muscles and adipose tissue [2]. This leads to symptoms such as pain, eyelid retraction, diplopia, and proptosis, which cause unexpected facial features and potentially progress to vision-threatening optic neuropathy [3].

Many quality-of-life studies of TED have been conducted in Europe and the United States, and the way symptoms appear seems to differ between the United States, Europe, and Asian countries [4-8]. Nevertheless, research exploring patient perspectives in Japan is limited. Most notably, no comprehensive quantitative study has examined health-related quality of life (HRQOL), psychosocial factors, or treatment satisfaction in Japanese patients with TED. Addressing this gap is crucial for developing appropriate therapeutic strategies and for improving clinical communication for TED patients.

The objective of this study was to assess HRQOL and psychosocial burdens in Japanese TED patients using patient-reported outcome measures. This effort aims to support a more holistic understanding of TED's impact and unmet patients' needs around the world.

## Methods

### Study design

The quantitative component used a cross-sectional, self-administered survey. Demographics, current symptoms, and treatment/surgery history were collected.

### Participant recruitment

Participants were recruited via a patient organization and collaborating ophthalmologists who distributed flyers with a link to the online form. Electronic informed consent was obtained; postal and telephone options were provided for accessibility.

### Instruments

Disease activity was defined as ongoing immunosuppressive therapy or ≥3 “present” items on the 7-item Clinical Activity Score. Severity followed European Group on Graves' Orbitopathy (EUGOGO) and was inferred from current management: mild (watchful waiting/supportive care only), moderate to severe (immunosuppression or surgery), and sight-threatening (urgent intervention) [9, 10].

Patient-reported outcomes were assessed using the Short-Form 12-Item Survey version 2 (SF-12v2) reported as norm-based T-scores (Japanese mean 50, SD 10; higher scores indicate better HRQOL) [11]. The original SF-12v2 consists of the 2-component model, which gives physical component summary (PCS) and mental component summary (MCS) scores, while SF-12v2 in Japanese consists of the 3-component model, which gives PCS, MCS, and role component summary (RCS) scores. This 3-component model was proposed by Ware and validated by Suzukamo et al [11]. Moreover, we utilized the Graves' Ophthalmopathy Quality of Life (GO-QOL) subscales (visual functioning, appearance; range 0-100; higher scores indicate better quality of life) [10, 12]; the Hospital Anxiety and Depression Scale (HADS; anxiety, depression; range 0-21; higher scores indicate worse symptoms) [13]; and the Client Satisfaction Questionnaire 8 (CSQ-8; range 8-32; higher scores indicate greater satisfaction with care) [14].

### Statistical analysis

Descriptive statistics were calculated for each variable. Symptom-presence comparisons used Welch's *t*-test with bias-corrected Hedges' g (95% confidence interval); interscale associations used Spearman's rank correlation. Missing data were handled as available cases for group tests and pairwise deletion for correlations. IBM SPSS Statistics version 28.0 (IBM Corp., Armonk, NY) was used. *P*-values < .05 were considered significant.

### Ethics

In accordance with the principles of the Declaration of Helsinki, the protocol was approved by the ASrid COI Management and ethical review committee (ap24032201). Participants received an explanation of the study from the researcher and submitted informed consent prior to participation. Data was anonymized; participation was voluntary with the option to withdraw. Participants received an honorarium, approved by the ethics committee, upon survey completion.

## Results

### Participant characteristics

A total of 56 individuals with TED completed the quantitative survey, of whom 44 (78.6%) were female (Table 1). The mean age was 48.8 years (SD = 12.1), with an average disease duration of 6.9 years (SD = 8.4). Based on symptom reporting and treatment status, 35.7% were classified as having active TED and 78.6% classified as having mild disease severity. None were classified as sight-threatening (very severe) per EUGOGO criteria [9].

**Table 1:** Basic characteristics (n = 56)

		n or mean	% or SD
Current age	(years)	48.8	12.1
Age of symptom onset	(years)	42.2	14.0
Age at definite diagnosis	(years)	42.4	13.9
Disease duration	(years)	6.9	8.4
Time since definite diagnosis	(months)	14.3	36.6
Gender	Male	12	21.4
	Female	44	78.6
Thyroid-related diseases	None	6	10.7
	Graves' disease	48	85.7
	Hashimoto's disease	5	8.9
Smoking history	Never smoked	35	63.6
	Former smoker	18	32.7
	Current smoker	2	3.6
Disease activity	Active phase	20	35.7
	Inactive phase	36	64.3
Severity	Mild	44	78.6
	Moderate	12	21.4
SF-12	PCS	47.6	11.1
	MCS	50.7	8.4
	RCS	39.5	14.0
GO-QOL	Visual functioning	82.6	20.8
	Appearance	65.8	26.6
HADS	Anxiety	6.9	4.9
	Depression	6.1	3.9
CSQ-8	Satisfaction with care	25.6	4.2

Missing data were excluded from analyses. Disease activity (active vs inactive) was determined using the Clinical Activity Score together with current treatment status. Severity was classified by investigators according to predefined study criteria (mild vs moderate).

Abbreviations: CSQ-8, Client Satisfaction Questionnaire 8; GO-QOL, Graves' Ophthalmopathy Quality of Life Questionnaire; HADS, Hospital Anxiety and Depression Scale; MCS, mental component summary; PCS, physical component summary; RCS, role/social component summary (SF-12); SF-12, Short-Form 12-Item Survey.

Using the SF-12v2 instrument, the mean PCS score was 47.6 (SD = 11.1) and the mean MCS score was 50.7 (SD = 8.4). On the other hand, the RCS score was particularly lower, at 39.5 (SD = 14.0), than the national normal (mean = 50). Interestingly, RCS was lower in patients with both active and chronic phases (respectively, n = 20, 32.6 ± 14.9; n = 36, 43.4 ± 12.0), although they were even lower in the active phase.

Using the GO-QOL questionnaire, the mean visual functioning score was 82.6 (SD = 20.8), and the appearance-related score was 65.8 (SD = 26.6). This showed a similar trend to the result of the SF-12v2. Anxiety and depression on the HADS averaged 6.9 (SD 4.9) and 6.1 (SD 3.9), respectively. Using the clinical cutoff of ≥11 points, 18 participants (32.1%) screened positive for anxiety and 12 (21.4%) for depression. The mean CSQ-8 score was 25.6 (SD = 4.2).

### Symptom-associated burden on HRQOL

Tables 2, 3, and 4 show group differences in psychological scale scores by current symptoms across various instruments. The most commonly reported symptom was ocular discomfort (76.8%), followed by dry eye, eye fatigue, diplopia, and proptosis.

**Table 2:** Differences in health-related QOL (SF-12) scores according to current symptoms (n = 56)

			PCS				MCS				RCS			
Symptom	Status	n	Mean	SD	*P*	g [95% CI]	Mean	SD	*P*	g [95% CI]	Mean	SD	*P*	g [95% CI]
Eye discomfort*	Have	43	47.3	10.5	.720	−0.11 [−0.72, 0.50]	50.2	8.4	.440	−0.24 [−0.86, 0.37]	38.9	14.5	.530	−0.20 [−0.81, 0.42]
	Not have	13	48.6	13.3			52.3	8.5			41.7	12.3		
Pain on eye movement*	Have	16	45.4	11.0	.357	−0.27 [−0.84, 0.30]	48.4	9.0	.200	−0.38 [−0.96, 0.20]	31.5	16.8	.006	−0.84 [−1.43, −0.25]
	Not have	40	48.5	11.2			51.6	8.1			42.8	11.4		
Redness of eyelid*	Have	9	41.0	11.8	.051	−0.72 [−1.46, 0.00]	50.2	6.4	.850	−0.07 [−0.78, 0.64]	37.3	11.3	.599	−0.19 [−0.89, 0.52]
	Not have	47	48.9	10.6			50.8	8.8			40.0	14.5		
Swelling of eyelid*	Have	22	46.2	12.5	.429	−0.22 [−0.74, 0.32]	49.4	7.8	.346	−0.26 [−0.79, 0.28]	33.5	15.1	.008	−0.74 [−1.29, −0.19]
	Not have	34	48.6	10.2			51.5	8.8			43.5	11.9		
Conjunctival redness*	Have	14	46.8	11.6	.754	−0.10 [−0.69, 0.51]	49.2	10.3	.455	−0.23 [−0.83, 0.37]	31.0	16.3	.007	−0.85 [−1.47, −0.23]
	Not have	42	47.9	11.1			51.2	7.8			42.4	12.0		
Conjunctival edema*	Have	5	50.4	10.0	.558	0.27 [−0.64, 1.18]	44.3	12.8	.075	−0.84 [−1.75, 0.04]	37.1	10.3	.686	−0.19 [−1.09, 0.72]
	Not have	51	47.4	11.3			51.3	7.8			39.8	14.3		
Swelling of tear sac*	Have	5	48.9	10.5	.796	0.12 [−0.79, 1.03]	48.3	5.4	.518	−0.30 [−1.21, 0.61]	18.6	15.7	<.001	−1.83 [−2.83, −0.85]
	Not have	51	47.5	11.3			50.9	8.6			41.6	12.1		
Symptoms fluctuate within a day	Have	9	51.2	9.8	.301	0.38 [−0.33, 1.09]	51.0	5.7	.910	0.04 [−0.66, 0.74]	31.9	18.8	.073	−0.66 [−1.37, 0.06]
	Not have	47	47.0	11.3			50.6	8.9			41.0	12.6		
Diplopia	Have	25	45.8	12.0	.261	−0.30 [−0.82, 0.22]	52.1	8.3	.249	0.31 [−0.82, 0.44]	39.0	15.4	.801	−0.07 [−0.59, 0.45]
	Not have	31	49.1	10.3			49.5	8.5			40.0	12.9		
Strabismu	Have	19	47.6	13.0	.994	<.01 [−0.55, 0.54]	51.8	6.3	.468	0.20 [−0.30, 0.63]	36.2	15.7	.201	−0.36 [−0.91, 0.19]
	Not have	37	47.6	10.2			50.1	9.3			41.3	12.9		
Upper eyelid retraction	Have	9	46.4	13.7	.720	−0.13 [−0.83, 0.58]	51.8	4.1	.669	0.15 [−0.55, 0.86]	27.5	15.3	.004	−1.08 [−1.81, −0.35]
	Not have	47	47.9	10.7			50.5	9.0			41.9	12.6		
Proptosi	Have	23	45.6	11.5	.256	−0.31 [−0.83, 0.22]	50.3	9.2	.792	−0.07 [−0.60, 0.46]	36.9	16.6	.241	−0.32 [−0.84, 0.21]
	Not have	33	49.1	10.8			50.9	7.9			41.4	11.7		
Photophobia	Have	21	44.5	10.4	.101	−0.45 [−0.99, 0.09]	50.5	5.4	.902	−0.03 [−0.57, 0.51]	40.4	16.0	.740	0.09 [−0.44, 0.62]
	Not have	35	49.5	11.2			50.8	9.9			39.1	12.8		
Epiblepharon	Have	9	50.5	10.4	.399	0.31 [−0.41, 1.01]	52.3	6.9	.530	0.23 [−0.48, 0.93]	38.0	14.3	.719	−0.13 [−0.85, 0.58]
	Not have	47	47.1	11.3			50.4	8.7			39.8	14.0		
Tearing	Have	11	50.4	11.7	.359	0.31 [−0.35, 0.96]	49.2	9.5	.511	−0.22 [−0.89, 0.43]	33.7	17.5	.120	−0.52 [−1.20, 0.14]
	Not have	45	47.0	11.0			51.1	8.2			41.0	12.8		
Vision loss after TED onset	Have	19	45.4	12.7	.288	−0.30 [−0.85, 0.25]	50.5	9.3	.925	−0.03 [−0.57, 0.52]	29.8	13.8	<.001	−1.20 [−1.79, −0.61]
	Not have	37	48.8	10.2			50.8	8.0			44.6	11.2		
Visual field defect	Have	2	29.4	17.0	.017	−1.75 [−3.17, −0.31]	56.2	11.2	.348	0.67 [−0.73, 2.07]	43.6	11.2	.680	0.29 [−1.12, 1.71]
	Not have	54	48.3	10.5			50.5	8.4			39.4	14.1		
Blurred vision	Have	4	40.9	16.5	.210	−0.65 [−1.66, 0.36]	49.2	1.1	.237	−0.19 [−1.19, 0.82]	31.7	5.2	.033	−0.60 [−1.61, 0.41]
	Not have	52	48.2	10.6			50.8	8.7			40.2	14.3		
Scotoma	Have	4	43.8	15.9	.476	−0.37 [−1.37, 0.64]	49.2	6.9	.717	−0.19 [−1.19, 0.82]	23.7	21.0	.017	−1.26 [−2.28, −0.22]
	Not have	52	47.9	10.8			50.8	8.6			40.8	12.8		
Color vision abnormality	Have	2	49.1	2.8	.850	0.14 [−1.28, 1.53]	46.0	3.6	.426	−0.57 [−1.99, 0.83]	53.0	9.5	.166	1.00 [−0.42, 2.40]
	Not have	54	47.6	11.3			50.9	8.5			39.1	13.9		

Missing data were excluded from analyses. Group differences were tested using Welch's *t*-test. g denotes Hedges' g.

Abbreviations: CI, confidence interval; GO-QOL, Graves' Ophthalmopathy Quality of Life Questionnaire; MCS, mental component summary; PCS, physical component summary; QOL, quality of life; RCS, role/social component summary (SF-12); SF-12, Short-Form 12-Item Survey; TED, thyroid eye disease.

^*^Clinical Activity Score components.

**Table 3:** Differences in health-related QOL (GO-QOL) and HADS scores according to current symptoms (n = 56)

			Health related QOL（GO-QOL）								HADS			
			Visual functioning				Appearance				Anxiety			
Symptom	Status	n	Mean	SD	*P*	g [95% CI]	Mean	SD	*P*	g [95% CI]	Mean	SD	*P*	g [95% CI]
Eye discomfort*	Have	43	80.7	21.3	.200	−0.41 [−1.02, 0.21]	64.4	27.0	.460	−0.23 [−0.84, 0.38]	7.1	5.0	.480	0.22 [−0.39, 0.84]
	Not have	13	89.2	18.1			70.7	25.7			6.0	4.7		
Pain on eye movement*	Have	16	71.8	23.4	.012	−0.76 [−1.34, −0.17]	49.6	26.9	.003	−0.91 [−1.52, −0.31]	8.3	5.0	.180	0.40 [−0.18, 0.97]
	Not have	40	87.0	18.2			72.3	23.9			6.3	4.8		
Redness of eyelid*	Have	9	72.4	25.1	.107	−0.59 [−1.30, 0.13]	56.3	25.2	.241	−0.43 [−1.13, 0.29]	9.1	5.9	.133	0.55 [−0.17, 1.26]
	Not have	47	84.6	19.5			67.7	26.7			6.4	4.6		
Swelling of eyelid*	Have	22	75.8	22.5	.048	−0.55 [−1.08, −0.01]	52.6	23.4	.002	−0.88 [−1.43, −0.32]	9.0	5.6	.014	0.75 [0.20, 1.30]
	Not have	34	87.0	18.6			74.4	25.3			5.5	3.9		
Conjunctival redness*	Have	14	69.7	24.0	.006	−0.87 [−1.49, −0.26]	47.3	28.8	.002	−0.99 [−1.64, −0.37]	9.1	6.7	.126	0.63 [0.02, 1.26]
	Not have	42	87.0	17.9			72.0	23.1			6.1	3.9		
Conjunctival edema*	Have	5	65.5	29.8	.053	−0.92 [−1.83, 0.01]	47.5	36.1	.107	−0.76 [−1.67, 0.16]	10.0	7.9	.134	0.70 [−0.22, 1.62]
	Not have	51	84.3	19.3			67.6	25.3			6.6	4.5		
Swelling of tear sac*	Have	5	75.9	16.5	.452	−0.35 [−1.26, 0.56]	33.8	16.9	.004	−1.40 [−2.34, −0.45]	8.6	5.5	.409	0.39 [−0.53, 1.29]
	Not have	51	83.3	21.2			69.0	25.4			6.7	4.9		
Symptoms fluctuate within a day	Have	9	83.0	14.1	.959	0.02 [−0.69, 0.72]	53.5	20.5	.129	−0.55 [−1.28, 0.16]	7.9	4.4	.495	0.25 [−0.46, 0.95]
	Not have	47	82.6	21.9			68.2	27.2			6.7	5.0		
Diplopia	Have	25	74.6	23.2	.011	−0.73 [−1.27, −0.19]	65.8	26.7	.980	−0.01 [−0.53, 0.52]	6.3	5.1	.433	−0.21 [−0.73, 0.31]
	Not have	31	89.1	16.2			65.9	27.0			7.3	4.8		
Strabismus	Have	19	73.2	17.9	.014	−0.71 [−1.27, −0.14]	61.8	23.5	.425	−0.22 [−0.78, 0.33]	7.6	5.7	.434	0.22 [−0.33, 0.77]
	Not have	37	87.5	20.7			67.9	28.2			6.5	4.4		
Upper eyelid retraction	Have	9	76.7	22.3	.353	−0.34 [−1.04, 0.37]	52.1	30.0	.091	−0.62 [−1.33, 0.10]	9.4	5.6	.083	0.63 [−0.08, 1.34]
	Not have	47	83.8	20.5			68.5	25.4			6.4	4.6		
Proptosis	Have	23	76.3	23.0	.055	−0.53 [−1.06, 0.01]	54.3	27.1	.006	−0.77 [−1.31, −0.22]	8.2	6.0	.122	0.46 [−0.08, 0.99]
	Not have	33	87.1	18.1			73.9	23.5			5.9	3.8		
Photophobia	Have	21	80.7	20.3	.593	−0.15 [−0.68, 0.39]	62.8	25.4	.512	−0.18 [−0.72, 0.36]	7.8	4.8	.263	0.31 [−0.23, 0.84]
	Not have	35	83.8	21.2			67.7	27.5			6.3	4.9		
Epiblepharon	Have	9	86.0	18.9	.599	0.19 [−0.52, 0.89]	63.9	24.0	.812	−0.09 [−0.79, 0.62]	5.9	5.3	.522	−0.23 [−0.94, 0.47]
	Not have	47	82.0	21.2			66.2	27.3			7.0	4.9		
Tearing	Have	11	80.0	28.2	.647	−0.15 [−0.80, 0.50]	63.6	29.2	.762	−0.10 [−0.76, 0.56]	8.9	6.2	.122	0.52 [−0.14, 1.18]
	Not have	45	83.3	18.9			66.4	26.3			6.4	4.5		
Vision loss after TED onset	Have	19	70.4	24.0	.005	−0.96 [−1.53, −0.38]	50.0	27.6	<.001	−0.98 [−1.55, −0.40]	8.4	5.7	.087	0.49 [−0.07, 1.04]
	Not have	37	88.9	15.9			74.0	22.4			6.1	4.3		
Visual field defect	Have	2	80.2	19.2	.868	−0.12 [−1.53, 1.27]	75.0	26.5	.625	0.35 [−1.06, 1.77]	6.5	0.7	.917	−0.07 [−1.47, 1.34]
	Not have	54	82.7	21.0			65.5	26.8			6.9	5.0		
Blurred vision	Have	4	71.7	23.5	.276	−0.56 [−1.57, 0.45]	64.1	20.0	.891	−0.07 [−1.09, 0.95]	8.8	5.6	.427	0.41 [−0.60, 1.41]
	Not have	52	83.5	20.6			66.0	27.2			6.7	4.9		
Scotoma	Have	4	74.3	22.3	.411	−0.42 [−1.43, 0.58]	40.6	14.9	.040	−1.03 [−2.05, −0.01]	13.0	5.4	.008	1.41 [0.37, 2.44]
	Not have	52	83.3	20.7			67.8	26.4			6.4	4.6		
Color vision abnormality	Have	2	89.3	15.2	.649	0.33 [−1.09, 1.72]	68.8	26.5	.877	0.11 [−1.28, 1.50]	4.5	3.5	.493	−0.49 [−1.88, 0.91]
	Not have	54	82.4	21.0			65.7	26.9			6.9	4.9		

Missing data were excluded from analyses. Group differences were tested using Welch's *t*-test. g denotes Hedges' g.

Abbreviations: CI, confidence interval; GO-QOL, Graves' Ophthalmopathy Quality of Life Questionnaire; HADS, Hospital Anxiety and Depression Scale; QOL, quality of life; TED, thyroid eye disease.

^*^Clinical Activity Score components.

**Table 4:** Differences in HADS and CSQ-8 scores according to current symptoms (n = 56)

			HADS				CSQ-8			
			Depression				Satisfaction with care			
Symptom	Status	n	Mean	SD	*P*	g [95% CI]	Mean	SD	*P*	g [95% CI]
Eye discomfort*	Have	43	6.1	3.6	.930	−0.03 [−0.64, 0.58]	25.4	4.1	.490	−0.22 [−0.83, 0.40]
	Not have	13	6.2	4.7			26.3	4.5		
Pain on eye movement*	Have	16	7.4	2.7	.133	0.45 [−0.14, 1.04]	24.3	5.0	.146	−0.43 [−1.01, 0.15]
	Not have	40	5.7	4.2			26.1	3.7		
Redness of eyelid*	Have	9	5.8	3.4	.760	−0.11 [−0.81, 0.59]	23.6	3.5	.110	−0.58 [−1.29, 0.13]
	Not have	47	6.2	4.0			26.0	4.2		
Swelling of eyelid*	Have	22	6.9	4.1	.236	0.32 [−0.21, 0.85]	23.8	3.9	.009	−0.74 [−1.29, −0.18]
	Not have	34	5.7	3.7			26.7	4.0		
Conjunctival redness*	Have	14	6.6	4.6	.636	0.15 [−0.45, 0.72]	24.5	4.9	.259	−0.35 [−0.95, 0.26]
	Not have	42	6.0	3.6			26.0	3.9		
Conjunctival edema*	Have	5	7.6	3.6	.382	0.41 [−0.50, 1.31]	23.6	4.0	.267	−0.52 [−1.43, 0.40]
	Not have	51	6.0	3.9			25.8	4.2		
Swelling of tear sac*	Have	5	8.2	2.6	.216	0.58 [−0.34, 1.49]	22.8	5.3	.118	−0.74 [−1.65, 0.19]
	Not have	51	5.9	3.9			25.9	4.0		
Symptoms fluctuate within a day	Have	9	6.1	1.6	.963	−0.01 [−0.71, 0.70]	24.1	4.5	.247	−0.42 [−1.13, 0.29]
	Not have	47	6.2	4.2			25.9	4.1		
Diplopia	Have	25	5.0	3.4	.055	−0.52 [−1.06, 0.01]	23.8	3.9	.003	−0.82 [−1.36, −0.27]
	Not have	31	7.0	4.0			27.1	3.9		
Strabismus	Have	19	4.9	2.8	.084	−0.49 [−1.04, 0.07]	23.9	3.9	.033	−0.62 [−1.18, −0.05]
	Not have	37	6.8	4.2			26.4	4.1		
Upper eyelid retraction	Have	9	7.2	4.6	.366	0.33 [−0.38, 1.03]	23.5	2.8	.126	−0.59 [−1.33, 0.16]
	Not have	47	5.9	3.7			26.0	4.3		
Proptosis	Have	23	6.4	4.0	.744	0.09 [−0.44, 0.62]	23.6	4.4	.002	−0.87 [−1.42, −0.32]
	Not have	33	6.0	3.9			27.0	3.5		
Photophobia	Have	21	5.8	2.6	.523	−0.15 [−0.68, 0.41]	25.3	3.3	.644	−0.13 [−0.67, 0.42]
	Not have	35	6.4	4.5			25.8	4.7		
Epiblepharon	Have	9	7.7	5.7	.200	0.47 [−0.25, 1.17]	26.6	5.2	.459	0.27 [−0.44, 0.97]
	Not have	47	5.9	3.4			25.4	4.0		
Tearing	Have	11	6.8	4.7	.523	0.21 [−0.44, 0.86]	24.8	3.5	.509	−0.23 [−0.91, 0.45]
	Not have	45	6.0	3.7			25.8	4.3		
Vision loss after TED onset	Have	19	6.6	3.8	.550	0.17 [−0.38, 0.71]	24.4	4.9	.131	−0.43 [−0.98, 0.13]
	Not have	37	5.9	3.9			26.2	3.7		
Visual field defect	Have	2	5.5	5.0	.813	−0.17 [−1.56, 1.22]	25.0	0.0	.839	−0.15 [−1.54, 1.25]
	Not have	54	6.2	3.9			25.6	4.3		
Blurred vision	Have	4	4.3	3.8	.314	−0.52 [−1.53, 0.49]	23.3	1.3	.247	−0.60 [−1.61, 0.41]
	Not have	52	6.3	3.9			25.8	4.3		
Scotoma	Have	4	5.8	3.9	.835	−0.11 [−1.11, 0.91]	23.8	1.0	.364	−0.47 [−1.47, 0.54]
	Not have	52	6.2	3.9			25.8	4.3		
Color vision abnormality	Have	2	4.5	0.7	.546	−0.43 [−1.85, 0.98]	25.0	1.4	.839	−0.15 [−1.56, 1.27]
	Not have	54	6.2	3.9			25.6	4.3		

Missing data were excluded from analyses. Group differences were tested using Welch's *t*-test. g denotes Hedges' g.

Abbreviations: CI, confidence interval; CSQ-8, Client Satisfaction Questionnaire 8; HADS, Hospital Anxiety and Depression Scale; TED, thyroid eye disease.

^*^Clinical Activity Score components.

The presence of symptoms such as pain with eye movement, eyelid swelling, strabismus, upper eyelid retraction, swelling of the caruncle, and proptosis was significantly inverse-associated with the RCS of the SF-12v2, visual or appearance/psychosocial scores of the GO-QOL, or CSQ-8 satisfaction score.

### Correlation analyses

Table 5 presents Spearman's correlation coefficients between psychosocial measures. Significant correlations emerged between PCS and GO-QOL visual functioning (ρ = 0.42). Disease duration is negatively correlated with GO-QOL appearance/psychosocial score (ρ = −0.34). Longer time to diagnosis is correlated with higher depression (ρ = −0.32) and lower mental HRQOL (ρ = −0.35). Moreover, anxiety is negatively correlated with satisfaction with care CSQ-8 (ρ = −0.36).

**Table 5:** Correlation matrix of psychosocial measures with age, duration of illness, and time to diagnosis (n = 56)

				A	B	C	D	E	F	G	H	I	J	K
Current age		A	*ρ*											
			*P*											
Disease duration		B	*ρ*	0.01										
			*P*	.969										
Time to definite diagnosis		C	*ρ*	−0.06	0.33									
			*P*	.710	.035									
SF-12	PCS	D	*ρ*	−0.23	−0.20	−0.01								
			*P*	.088	.141	.948								
	MCS	E	*ρ*	−0.02	−0.14	−0.35	−0.05							
			*P*	.904	.298	.026	.732							
	RCS	F	*ρ*	−0.13	−0.08	−0.27	0.09	0.08						
			*P*	.356	.563	.092	.498	.537						
GO-QOL	Visual functioning	G	*ρ*	−0.36	−0.09	−0.08	0.42	0.00	0.42					
			*P*	.006	.534	.639	.001	.978	.001					
	Appearance	H	*ρ*	0.08	−0.34	−0.18	0.07	0.34	0.40	0.21				
			*P*	.554	.012	.250	.588	.010	.002	.117				
HADS	Anxiety	I	*ρ*	0.14	0.19	0.18	−0.25	−0.44	−0.52	−0.24	−0.43			
			*P*	.319	.169	.273	.062	<.001	<.001	.073	<.001			
	Depression	J	*ρ*	−0.07	0.08	0.32	−0.18	−0.37	−0.52	−0.15	−0.24	0.58		
			*P*	.595	.570	.042	.194	.006	<.001	.280	.073	<.001		
CSQ-8	Satisfaction with care	K	*ρ*	−0.30	−0.15	−0.05	0.07	0.24	0.25	0.25	0.35	−0.36	−0.07	
			*P*	.029	.289	.760	.596	.078	.062	.063	.009	.006	.601	

Missing data were excluded from analyses. Spearman's rank-order correlations (ρ) were used to assess significance.

Abbreviations: CSQ-8, Client Satisfaction Questionnaire 8; GO-QOL, Graves' Ophthalmopathy Quality of Life Questionnaire; HADS, Hospital Anxiety and Depression Scale; MCS, mental component summary; PCS, physical component summary; RCS, role/social component summary (SF-12); SF-12, Short-Form 12-Item Survey.

## Discussion

This is the first nationwide study in a Japanese population using validated instruments (eg, SF-12v2 and CSQ-8) to quantify these effects. Given that this survey was conducted prior to the approval of teprotumumab in Japan (September 2024), the findings offer critical insights into real-world unmet patients' needs under conventional medical treatment paradigms.

Among the SF-12v2 subscales, while the PCS and MCS scores were near the Japanese population norms (50 ± 10), the RCS was markedly reduced (39.5 ± 14.0). As RCS mainly consists of factors about “social functioning,” “role physical,” and “role emotional” [11], this pattern suggests that TED substantially restricts patients' ability to fulfill social roles, likely due to the visible facial changes and unpredictable disease course. Interviews with TED patients in Europe also indicated social withdrawal and work disability [15, 16]. These are consistent with a hypothesis that the RCS would be inversely related to the number of days of work missed per year for health-related reasons [11]. It has been shown that patients with TED have a large social burden, and it is important that we were able to quantify it as RCS in Japan.

Our findings also revealed that GO-QOL appearance-related scores were similarly impaired across the world (62.3 in the United States, 71.2 in Germany, 54.5 in Australia, 61.9 in Korea, 54.5 in Taiwan, and 65.8 in our study) [4-8], whereas visual functioning scores were better in the Japanese sample (58.6 in the United States, 72.5 in Germany, 59.0 in Australia, 73.7 in Korea, 58.4 in Taiwan, 82.6 in our study). This may reflect anatomical and cultural differences. Craniofacial skeletal structure in Asian populations (eg, shallower orbits, wider intercanthal distance) may attenuate the visual consequences of proptosis, eyelid swelling, and retraction [17, 18]. In addition, Japanese patients worry more about what other people think than Western people, although the changes in symptoms are modest. However, the psychological and appearance-related distress appears to be universally significant, highlighting the central role of psychosocial burden in TED regardless of ethnicity. Lin et al [8] also mentioned characteristics of Asian TED patients using a cohort including sight-threatening disease, but our study confirmed this without sight-threatening disease.

Compared with participants without symptoms, those with pain in eye movement, eyelid swelling, strabismus, upper eyelid retraction, or proptosis had significantly lower SF-12v2 RCS, GO-QOL (visual functioning and appearance), and CSQ-8 scores. Smith et al [19] reported that all these symptoms bothered US patients, as in our cohort. On the other hand, swelling of the caruncle was not mentioned as a symptom bothering patients in the United States [19] but affected the RCS of the SF12v2 and the psychosocial score of the GO-QOL in our study. These findings suggest that visible disfigurement—not just functional symptoms—is a key driver of quality-of-life impairment in TED.

Our study also revealed that there was a correlation between time to diagnosis and depression/mental HRQOL. Moreover, the CSQ-8 is negatively correlated with anxiety. This could be due to the characteristics of TED, which is a rare and progressive disease. TED covers both internal medicine and ophthalmology, making a definitive diagnosis difficult. As time to diagnosis is longer, TED is progressive with worsening symptoms over time, which has a psychological impact. To address this, it is important to raise disease awareness and establish a referral network. Efforts at the EUGOGO may be useful as a reference [20].

Limitations of this study include reliance on self-reported disease activity and severity, the exclusion of patients with very severe TED, and the lack of multivariate adjustment for potential confounders. However, the sample represents a real-world cross-section of patients under conventional treatment in Japan.

By integrating qualitative findings from in-depth patient interviews, our future analyses will employ mixed-methods triangulation to ascertain how symptoms like proptosis, care pathways, interpersonal experiences, and sociocultural expectations collectively shape HRQOL. Crucially, this integration is anticipated to contribute not only to the identification of therapeutic targets but also to the formulation of system-level interventions aimed at enhancing continuity of care and improving the overall patient experience in TED.

## Conclusion

Japanese patients with TED experience significant reductions in role functioning and psychosocial quality of life, especially during active disease conditions with symptomatic burden. These results underline the importance of integrative and comprehensive psychosocial support and patient-centered communication in TED management.

## Data Availability

The datasets generated during the current study are owned by ASrid. These are not publicly available in order to avoid identifying individuals, as TED is a rare disease.
